# Parents’ views on sex education in schools: How much do Democrats and Republicans agree?

**DOI:** 10.1371/journal.pone.0180250

**Published:** 2017-07-03

**Authors:** Leslie Kantor, Nicole Levitz

**Affiliations:** Planned Parenthood Federation of America, New York, New York, United States of America; Pennsylvania State University, UNITED STATES

## Abstract

More than 93 percent of parents place high importance on sex education in both middle and high school. Sex education in middle and high school is widely supported by parents regardless of their political affiliation. Using data from a large diverse sample of 1,633 parents of children aged 9 to 21 years, we examined whether views on sex education differed by parents’ political affiliation. More than 89 percent of parents that identified as Republicans or Democrats support including a wide range of topics in sex education including puberty, healthy relationships, abstinence, sexually transmitted diseases (STDs) and birth control in high school. In middle school, 78 percent or more of both parents that identified as Republicans and Democrats support the inclusion of those topics. Controlling for key demographic factors, parents that identified as Democrats are more likely than those that identified as Republicans to support the inclusion of the topics of healthy relationships, birth control, STDs, and sexual orientation in both middle and high school. However, a strong majority of Republican parents want all these topics included in sex education. Sex education which includes a broad set of topics represents an area of strong agreement between parents of both political parties.

## Introduction

The vast majority of Americans support comprehensive sex education in public schools [1–4]. However, the type of sex education received in US public schools varies greatly. The Centers for Disease Control and Prevention identifies sixteen HIV, STD or pregnancy prevention topics that should be included in school-based sex education [5]. States report a wide range in the percentage of topics covered from 21.0% to 89.5% [5].

In past surveys, more than ninety percent of Americans over age eighteen felt sex education was “very important” or “somewhat important” to include in public school curricula [4]. Ninety-three percent (93 percent) of adults supported teaching sex education in high school and 84 percent in middle school, with some differences in support by geographic region [6]. State-specific data available from Minnesota, California, North Carolina, South Carolina, Delaware, Florida, Kansas and Indiana demonstrates support for including information on birth control and condoms in sex education [1, 7–8]. Studies also show that most adults believe middle school is the most appropriate time to start sex education [1, 7–8].

Several surveys have specifically examined parents’ views on sex education and these also show very high support for sex education in schools [9–13]. Very few parents believe sex education should not be taught in schools [10]. Parents, regardless of race/ethnicity, income or age, support comprehensive sex education [9–10]. However, parents report a large gap between what they want and what is actually being offered to their children in schools [13]. In a study in North Carolina, 91 percent of parents said they wanted sex education taught in schools but only 67 percent said it was being taught [13]. Generally speaking, younger age, African American race, attending religious services less frequently, higher education levels, lower household income, and being more politically liberal increase the odds of parents supporting comprehensive sex education [9, 13]. However, all parents are highly supportive of sex education in schools.

Among parents, there is strong support for including a variety of topics in sex education. In previous studies, about 70 percent of parents support the inclusion of birth control, HIV/AIDS, and preventing pregnancy [10, 12–13]. In a sample of immigrant parents, many topics such as transmission and prevention of HIV/AIDS, transmission and prevention of STIs and how to prevent sexual abuse were supported by more than 80 percent of parents and only abortion, anal sex, oral sex and masturbation had support from fewer than 80 percent of parents [2]. A study comparing the views of parents in zip codes with a high incidence of teen pregnancy to those living areas of lower incidence, found that parents in the high incidence areas were significantly more likely to say it was “very important” to include the topics of birth control and sexual orientation in sex education [12].

A review of the literature identified only two existing studies evaluating the association between political affiliation and sex education [11, 14], and only a few others looking at the role of political conservativeness or liberalism and views on sex education among parents [9–10, 15]. This study assesses the association between political affiliation and support for sex education using a large, diverse sample of parents from across the United States. We examined both general assessments of the perceived importance of sex education in schools and support for including particular topics in middle and high school, and examined differences between parents that identified as Republicans and Democrats.

## Methods

### Ethics statement

This study analyzed secondary, de-identified data that was collected and provided by GfK. The paper describes the consent and assent procedure utilized by Gfk in for parent and child participants. For the present study, we only used data from parents. GfK indicates, "The KnowledgePanel recruitment and empanelment process is designed to comply with CAN-SPAM Act and Council of American Survey Research Organizations guidelines. Further, our policies conform to participant treatment protocols outlined by the federal Office Management and Budget, following guidelines from the Belmont Report. Survey responses are confidential; personally identifying information is never revealed to clients or other external parties without explicit respondent approval and a client-signed nondisclosure agreement.” When surveys are assigned to KnowledgePanel panel members, they are notified in their password-protected email account that a survey is available for completion. Surveys are self-administered and accessible any time of day for a designated period. Participants can complete a password-protected survey only once. Members may withdraw from the panel at any time, and continued provision of the web-enabled device (e.g., laptop or netbook) and Internet service is not contingent on completion of any particular survey.

### Respondents

Parents were surveyed in July, 2014 by Gfk, Inc. Gfk has constructed a large and diverse panel of adults in the United States, who were recruited using a combination of random digit dial phone techniques and address-based sampling. More information on the construction of the overall Gfk panel is available at: http://www.gfk.com/en-us

Parents for this study were sampled from the broader Gfk panel using e-mail invitations and asked to consent for themselves and one of their children between the ages of 9 and 21, who was then asked to assent into the study. For White parents, a random selection of parents from the panel was invited. All Latino and African American parents in the Gfk panel were invited to participate in this study.

A total of 1,633 parents completed the surveys. Seven hundred and eleven (711) parents were White, 300 were African American and 652 were Latino. One thousand eighty-one (1,081) mothers and 582 fathers completed the survey. The survey was conducted online although, as noted previously, the panel was recruited using random digit dialing and address based sampling. Only parent data were analyzed for this study. If the participant indicated they were undecided or were Independent of a political party, they were removed from this analysis. Seventy one people were removed leaving a total N of 1,592.

### Measures

The parent questionnaire contained 91-items. The median survey completion time was 17 minutes. Parents were asked “How important do you think it is to have sex education in middle school?” and the same item for high school. Support was measured using the following answer choices: “very important,”, “somewhat important,” “not important,” “should not be taught in middle/high school” and “should not be taught in school at all.”

To assess support for the inclusion of topics, there were a series of dichotomous yes-no questions: “Should sex education in school include the following [topic]?” The topics included were puberty, healthy relationships, abstinence, birth control methods, sexually transmitted diseases, and sexual orientation. Parents were asked about whether they supported the inclusion of these topics in middle and high school.

For political affiliation, participants chose between seven discriminations, strong Republican/Democrat, not strong Republican/Democrat, leans Republican/Democrat, or Undecided/Independent/Other. Undecided/Independent/Other was removed completely as this study sought to examine the distinctions between Democrats and Republicans in support for sex education. We then examined the sample size in each of remaining six discriminations and found each had at least 297 individuals. We explored each of the possible ways of collapsing the political affiliation categories and found that the significance of the results did not differ, so we collapsed the three levels of Republican and Democrat into a dichotomous variable to facilitate interpretation of the data and its applicability.

## Results

See Table 1 for key demographics of the parent sample as a whole and by political affiliation. The sample had about 20 percent more Democrats in it than Republicans. Level of educational attainment and employment status was comparable across political affiliations. There was a roughly equal percentage of male and female Republicans, while 70 percent of Democrats were female. A higher percentage of Republicans reported being White and married compared to Democrats (see Table 1). These demographic factors are associated with differences in views on sex education. Generally, White individuals, married individuals and men are less likely to support sex education compared to people of color, unmarried individuals, and women. Despite this, we still see high levels of support no matter the group.

**Table 1:** Demographics of sample parents.

Demographics	*All*	*Democrats*	*Republicans*
***Sex***			
**Female**	65.0% (n = 1,081)	70.0% (n = 694)	55.5% (n = 333)
**Male**	35.0% (n = 582	30.0% (n = 298)	44.5% (n = 267)
***Median household income range***	$40,000–$49,000	$40,000–$49,000	$60,000–$79,000
***Marital Status***			
**Married**	70.1% (n = 1,166)	61.2% (n = 608)	84.5% (n = 507)
**Single**	29.9% (n = 497)	38.7% (n = 384)	16.2% (n = 97)
***Employment***			
**Employed**	66.6% (n = 1,108)	68.7% (n = 633)	71.7% (n = 430)
**Unemployed**	33.3% (n = 555)	39.0% (n = 359)	28.3% (n = 170)
***Education***			
**Some College**	63.9% (n = 1,063)	64.0% (n = 635)	66.5% (n = 399)
**No College**	36.1% (n = 600)	36.0% (n = 357)	33.5% (n = 201)
***Race/Ethnicity***			
**Black, Non-Hispanic**	18.0% (n = 300)	26.3% (n = 261)	4.5% (n = 27)
**Latino**	39.2% (n = 652)	44.6% (n = 442)	28.6% (n = 172)
**White, Non-Hispanic**	42.8% (n = 711)	29.1% (n = 289)	66.8% (n = 401)
***Political Affiliation***			
**Democrat**	59.7% (n = 992)		
**Republican**	37.7% (n = 600)		
**Independent**	3.4% (n = 56)		

### Overall importance of sex education in schools

In middle school, 74.9 percent of parents (n = 1,240) felt it was very important to have sex education, and an additional 18.6 percent (n = 308) felt it was somewhat important (See Table 2). Only 2.7 percent (n = 44) felt sex education should not be taught at all, 2.6 percent (n = 43) felt it should not be taught in middle school and 1.3 percent (n = 21) felt it was not important to have sex education in middle school.

**Table 2:** Importance of sex education overall.

	Middle School			High School		
	Overall	Republican	Democrat	Overall	Republican	Democrat
**Very Important**	74.9%	64.0%	82.0%	86.0%	77.2%	92.0%
**Somewhat Important**	18.6%	26.1%	14.1%	10.0%	16.7%	6.5%
**Not Important**	1.3%	1.4%	0.9%	1.4%	1.6%	0.9%
**Should not be taught in**	2.6%	3.2%	2.2%	0.5%	1.1%	0.0%
**Should not be taught at all**	2.7%	6.2%	0.5%	2.2%	4.6%	0.8%
**Mean (SD)**	1.4 (0.860)	1.6 (1.095)	1.3 (.640)	1.2 (0.703)	1.4 (0.947)	1.1 (.458)

Note: Median and mode for both middle and high school was 1 (e.g. “very important”).

In high school, 86.0 percent (n = 1,423) of respondents felt it was very important to have sex education, 10.0 percent (n = 165) felt it was somewhat important, and 1.4 percent (n = 23) felt it was not important. The percentage of parents who felt sex education should not be taught at all remained about the same as for middle school (2.2 percent), but there were fewer parents (n = 8) who felt it shouldn’t be taught in high school, as compared to middle school.

### Differences in importance of sex education in schools by political affiliation

Eighty-two percent of parents who identified as Democrats felt it was very important to include sex education in middle school compared to 64.0 percent of parents who identified as a Republican. Ninety-two percent of parents who were Democrats felt it was very important to have sex education in high school compared to 77.2 percent of Republican parents. Although collectively Democrats placed a higher importance on sex education in both middle and high school than Republicans, a majority of Republican parents found it very important to include sex education in both middle and high school.

When controlling for race/ethnicity, income, employment, marital status, gender, and education, we found that identifying as a Republican slightly increased the odds of placing importance on sex education in middle school (B = 0.12, p <.001) and high school (B = 0.09, p <.001). However, because the odds are close to zero, there was essentially no difference by political affiliation. Regardless of political affiliation, sex education was of high importance to parents.

### Overall support for inclusion of sex education topics

Overall, parents were very supportive of including all six key sex education topics included in the survey in both middle and high school: puberty, healthy relationships, abstinence, birth control, sexually transmitted diseases (STDs) and sexual orientation (see Table 3). There was more support for including all of the topics in high school compared to middle school, except for puberty which had slightly greater support for inclusion in middle school. For high school, all six sex education topics, except sexual orientation, had over 94.0 percent parental support. Eighty-five percent of parents supported including sexual orientation in high school sex education.

**Table 3:** Support by political affiliation for sex education topics.

	Middle School			High School		
Topic	Overall	Republican	Democrat	Overall	Republican	Democrat
Puberty	97.7% (n = 1,612)	97.2% (n = 580)	98.6% (n = 971)	95.1% (n = 1,581)	94.1% (n = 562)	97.3% (n = 957)
Healthy Relationships	92.3% (n = 1,519)	89.1% (n = 530)	94.7% (n = 931)	96.1% (n = 1,581)	92.8% (n = 553)	98.6% (n = 969)
Abstinence	94.8% (n = 1,541)	94.7% (n = 558)	95.6% (n = 927)	96.4% (n = 1,573)	96.0% (n = 566)	97.3% (n = 948)
Birth Control	86.3% (n = 1,418)	78.9% (n = 468)	91.5% (n = 899)	94.4% (n = 1,555)	89.3% (n = 532)	97.9% (n = 964)
STDs	96.2% (n = 1,589)	94.0% (n = 562)	98.2% (n = 968)	98.1% (n = 1,616)	96.8% (n = 577)	99.3% (n = 978)
Sexual Orientation	78.0% (n = 1,281)	64.7% (n = 383)	87.0% (n = 855)	85.0% (n = 1,390)	74.9% (n = 444)	92.0% (n = 899)

Note: Median and mode for all topics for both middle and high school was 1 “very important.”

### Differences in views on inclusion of topics by political affiliation

Similar percentages of Republicans and Democrats wanted healthy relationships, STDs, puberty and abstinence included in both middle and high school (see Table 3). However, there were differences in support by political affiliation for two topics: sexual orientation and birth control. For sexual orientation, 92.0 percent of Democrats said it should be included in high school sex education programs compared to 74.9 percent of Republicans. For birth control, 97.9 percent of Democrats said it should be included in high school sex education compared to 89.3 percent of Republicans.

When controlling for race/ethnicity, income, employment, marital status, gender, and education, we found that identifying as a Democrat significantly increased the odds of parents supporting the inclusion of birth control, STIs, healthy relationships, and sexual orientation in both middle and high school (see Table 4).

**Table 4:** Odds ratios for sex education topics by political affiliation.

Topic	OR	SE	95% CI	Significance
**Puberty**				
MS	1.8	0.407	(0.8, 3.9)	p = 0.159
HS	2.0*	0.291	(1.1, 3.5)	p = 0.021
**Healthy Relationships**				
MS	2.5*	0.226	(1.6, 3.9)	p<0.001
HS	6.6*	0.351	(3.3, 13.2)	p<0.001
**Abstinence**				
MS	0.9	0.266	(0.6,1.6)	p = 0.800
HS	1.4	0.314	(0.8, 2.6)	p = 0.290
**Birth Control**				
MS	2.5*	0.171	(1.8, 3.5)	p<0.001
HS	6.0*	0.285	(3.4, 10.5)	p<0.001
**STDs**				
MS	2.6*	0.325	(1.4, 2.6)	p = 0.003
HS	7.1*	0.509	(2.6, 19.1)	p<0.001
**Sexual Orientation**				
MS	3.5*	0.144	(2.6, 4.6)	p<0.001
HS	3.9*	0.172	(2.8, 5.5)	p<0.001

* p <.05

Note: Republicans are the reference group and we controlled for parent marital status, household income, parent employment status, parent education, parent sex, and parent race/ethnicity.

Parents of both political affiliations generally supported the inclusion of birth control in sex education. However, parents that identified as Democrats had greater odds of supporting the inclusion of birth control in both middle and high school. The odds of a Democrats supporting inclusion of birth control in sex education was greater for high school (OR = 6.0, 95% CI (3.4, 10.5)) than middle school (OR = 2.5, 95% CI (1.8, 3.5)).

STDs had the widest difference in odds ratios from middle to high school of all the sex education topics, but this was due to the presence of a large confidence interval. Democrats had greater odds of supporting the inclusion of STDs in both middle and high school. The odds of a Democrat supported the inclusion of birth control in sex education was greater for high school (OR = 7.056, 95% CI (2.6, 19.1)) than middle school (OR = 2.56, 95% CI (1.4, 2.6)).

Healthy relationships followed a familiar pattern with greater odds of having supported the topic’s inclusion among Democrats for high school compared to middle school. Democrats had greater odds than Republicans of supporting the inclusion of healthy relationships in both middle and high school. The odds a Democrat supported the inclusion of healthy relationships in sex education was greater school (OR = 6.6, 95% CI (3.3, 13.2)) than middle school (OR = 2.5, 95% CI (1.6, 3.9)) to high.

Sexual orientation had a different pattern than the other topics. In general, for all topics the inclusion of topics was more highly supported in high school than in middle school among Democrats. For sexual orientation, the odds of support stayed the same between middle and high school. Parents that identify as Democrats had greater odds of supporting the inclusion of sexual orientation in both middle and high school compared to Republican parents. The odds a Democrat supported the inclusion of sexual orientation in sex education compared to Republic parents stayed was the same for middle (OR = 3.5, 95% CI (2.6, 4.6)) and high school (OR = 3.9, 95% CI (2.8, 5.5)).

Abstinence was the only topic where there was no significant difference in support by political affiliation in both middle and high school.

Puberty was the only topic on which there was no difference by political affiliation in middle school but there was a significant difference in high school. Parents that identified as Democrats were more likely than Republicans to want puberty included in high school sex education (OR = 1.955, 95% CI (1.1,3.5)). All other topics (besides abstinence) had significant differences in odds by political affiliation for both middle and high school.

## Discussion

Regardless of political affiliation, parents overwhelmingly report that sex education in both middle and high school is important and want sex education to include a variety of topics such as puberty, healthy relationships, abstinence, birth control, and STDs.

Few previous studies have examined the role of political affiliation in support for sex education. Both parents that identify as Democrats and parents that identify as Republicans believe sex education is important in both middle and high school. In fact, Republican parents place slightly higher overall importance on sex education in both middle and high school, controlling for other factors. One limitation of the importance item is that respondents may have interpreted sex education differently. However, the surveys items related to particular topics illustrate that the vast majority of parents want a variety of topics covered in sex education, including puberty, healthy relationships, abstinence, birth control methods, sexually transmitted diseases, and sexual orientation. When controlling for demographic differences, the odds of parents that identify as Democrats wanting an individual topic included in sex education are greater for all topics but abstinence, but a large majority of Republicans support inclusion of all of the topics. In general, there is greater support for including all of the topics in high school compared to middle school. Republicans and Democrats place almost equal value on the importance of sex education controlling for demographic factors.

This research adds to a growing body of evidence that sex education which includes a wide range of topics, often referred to as comprehensive sex education, is supported by the vast majority of parents, including both Democrats and Republicans. Policy makers, school administrators and teachers should be aware that parents believe providing middle and high school students with sex education is important and that they want numerous topics covered.

## Supporting information

S1 File Survey. (DOCX)

S2 File Data set. (SAV)

## References

[1] Herrman JW, Solano P, Stotz L, McDuffie MJ. 2013. Comprehensive sexuality education: A historical and comparative analysis of public opinion. Am J Sex Educ. 2013; 8(3): 140–159. doi: 10.1080/15546128.2013.828342

[2] Heller HR, Johnson HL. Parental opinion concerning school sexuality education in a culturally diverse population in the USA. Sex Edu. 2013; 13(5): 548–559. doi: 10.1080/14681811.2013.775063

[3] Bleakley A, Hennessy M, Fishbein M. Public opinion on sex education in US schools. Arch Pediatr Adolesc Med. 2006; 160(11): 1151–1156. doi: 10.1001/archpedi.160.11.1151 17088519 10.1001/archpedi.160.11.1151

[4] National Public Radio (NPR), Kaiser Family Foundation, and Kennedy School of Government.Sex Education in America: General public/parents survey. 2004. http://www.npr.org/programs/morning/features/2004/jan/kaiserpoll/publicfinal.pdf.

[5] Demissie Z, Brener ND, McManus T, Shanklin S, Hawkins J, Kann L. School health profiles 2014: Characteristics of health programs among secondary schools. US Department of Health and Human Services, 2015. https://www.cdc.gov/healthyyouth/data/profiles/pdf/2014/2014_profiles_report.pdf

[6] Landry DJ, Darroch JE, Singh S, Higgins J. Factors associated with the content of sex education in U.S. public secondary schools. Perspect Sex Reprod Health. 2007; 35(6):261–269. 10.1363/psrh.35.261.03 14744658

[7] Howard-Barr E, Moore MJ, Weiss JA, Jobli E. Public opinion toward sexuality education: Findings among one south Florida county. Am J Sex Educ. 2011; 6(2): 176–191. doi: 10.1080/15546128.2011.571955

[8] Lewis RK, Paine-Andrews A, Custard C, Stauffer M, Harris K, Fisher J. Are parents in favor or against school-based sexuality education? A report From the Midwest. Health Promot Pract. 2001; 2(2): 155–161.

[9] Constantine NA, Jerman P, Huang AX. California parents’ preferences and beliefs regarding school-based sex education policy. Perspect Sex Reprod Health. 2007; 39(3): 167–175. doi: 10.1363/3916707 17845528 10.1363/3916707

[10] Eisenberg ME, Bernat DH, Bearinger LH, Resnick MD. Support for Comprehensive sexuality education: Perspectives from parents of school-age youth. J Adolesc Health. 2008; 42: 352–359. doi: 10.1016/j.jadohealth.2007.09.019 18346660 10.1016/j.jadohealth.2007.09.019

[11] Alton FL, Valois RF, Oldendick R, Drane JW. Public opinion on school-based sex education in South Carolina. Am J Sex Educ. 2009; 4(2): 116–138. doi: 10.1080/15546120903001381

[12] Millner V, Mulekar M, Turrens J. Parents beliefs regarding sex education for their children in southern Alabama public schools. Sex Res Social Policy. 2015; 12: 101–109.

[13] Ito KE, Gizlice Z, Owen-O’Dowd J, Foust E, Leone PA, Miller WC. Parent opinion of sexuality education in a state with mandated abstinence education: Does policy match parental preference? J Adolesc Health. 2006; 39: 634–641. doi: 10.1016/j.jadohealth.2006.04.022 17046498 10.1016/j.jadohealth.2006.04.022

[14] Howard-Barr EM, Moore MJ. Florida residents' preferred approach to sexuality education. Am J Sex Educ. 2007; 2(3): 27–37. doi: 10.1300/J455v02n03_03

[15] Bleakley A, Hennessy M, Fishbein M. Predicting preferences for types of sex education in U.S. schools. Sex Res Social Policy. 2010; 7: 50–57. doi: 10.1007/s13178-010-0008-z

