# Supplementary material for: Parents’ views on sex education in schools: How much do Democrats and Republicans agree?
Source: PLoS One. 2017 Jul 3;12(7):e0180250. doi: 10.1371/journal.pone.0180250 (PMC5495344; doi:10.1371/journal.pone.0180250)
Supplement: S1 File — (DOCX) [file pone.0180250.s001.docx]

**Parents’ Views on Sex Education in Schools: How much do Democrats and Republicans Agree?**

P1. How important do you think it is to have sex education in middle school?

1. Very important
2. Somewhat important
3. Not important
4. Sex education should not be taught in middle school
5. Sex education should not be taught in school at all

P2. Should sex education in middle school include the following topics?

| Yes | No |
| --- | --- |

1. Abstinence
2. Puberty and the physical, social and emotional changes that take place during the teen years
3. Birth control methods
4. Sexually transmitted diseases, including HIV
5. Healthy and unhealthy romantic relationships
6. Sexual orientation (e.g. information about being straight, gay, lesbian, or bisexual)

P3. How important do you think it is to have sex education in high schools?

1. Very important
2. Somewhat important
3. Not important
4. Sex education should not be taught in high school
5. Sex education should not be taught in school at all

P4. Should sex education in high school include the following topics?

| Yes | No |
| --- | --- |

1. Abstinence
2. Puberty and the physical, social and emotional changes that take place during the teen years
3. Birth control methods
4. Sexually transmitted diseases, including HIV
5. Healthy and unhealthy romantic relationships
6. Sexual orientation (e.g. information about being straight, gay, lesbian, or bisexual)
